# ASB20123: A novel C-type natriuretic peptide derivative for treatment of growth failure and dwarfism

**DOI:** 10.1371/journal.pone.0212680

**Published:** 2019-02-22

**Authors:** Naomi Morozumi, Takafumi Yotsumoto, Akira Yamaki, Kazunori Yoshikiyo, Sayaka Yoshida, Ryuichi Nakamura, Toshimasa Jindo, Mayumi Furuya, Hiroaki Maeda, Yoshiharu Minamitake, Kenji Kangawa

**Affiliations:** 1 Asubio Pharma Co., Ltd., Kobe, Japan; 2 Daiichi Sankyo Co., Ltd., Tokyo, Japan; 3 National Cerebral and Cardiovascular Center Research Institute, Osaka, Japan; Max Delbruck Centrum fur Molekulare Medizin Berlin Buch, GERMANY

## Abstract

C-type natriuretic peptide (CNP) and its receptor natriuretic peptide receptor B (NPR-B) are physiological potent positive regulators of endochondral bone growth; therefore, the CNP/NPR-B signaling pathway is one of the most promising therapeutic targets for treating growth failure and dwarfism. In this article, we summarized the pharmacological properties of a novel CNP analog peptide ASB20123 as a therapeutic agent for short stature. ASB20123, one of the CNP/ghrelin chimeric peptides, is composed of CNP(1–22) and human ghrelin(12–28, E17D). Compared to CNP(1–22), ASB20123 showed similar agonist activity for NPR-B and improved biokinetics with a longer plasma half-life in rats. In addition, the distribution of ASB20123 to the cartilage was higher than that of CNP(1–22) after single subcutaneous (*sc*) injection to mice. These results suggested that the C-terminal part of ghrelin, which has clusters of basic amino acid residues and a BX7B motif, might contribute to the retention of ASB20123 in the extracellular matrix of the growth plate. Multiple *sc* doses of ASB20123 potently stimulated skeletal growth in rats in a dose-dependent manner, and *sc* infusion was more effective than bolus injection at the same dose. Our data indicated that high plasma levels of ASB20123 would not necessarily be required for bone growth acceleration. Thus, pharmaceutical formulation approaches for sustained-release dosage forms to allow chronic exposure to ASB20123 might be suitable to ensure drug effectiveness and safety.

## Introduction

C-type natriuretic peptide (CNP) is a member of the natriuretic peptide (NP) family that also includes atrial natriuretic peptide (ANP) and brain natriuretic peptide (BNP) [1]. ANP and BNP are predominantly produced in the atria and ventricles of heart and are suggested to play an important role in the regulation of cardiovascular homeostasis [2]. Additionally, they have been developed as diagnostic tools and therapeutic drugs for cardiac failure [3, 4]. However, CNP is expressed in various tissues, such as the central nervous system, reproductive tract, bone, and endothelium of blood vessels. CNP mainly acts as an autocrine/paracrine factor [5]. In particular, CNP and its receptor natriuretic peptide receptor-B (NPR-B) signaling is a pivotal stimulator of endochondral bone growth [6, 7], and CNP or its analogue could be one of the most expecting therapeutic approaches to short statue patients, such as achondroplasia [8]. CNP(1–22) is a major endogenous molecular form of CNP in the plasma. Exogenous CNP(1–22) was rapidly cleared from the circulation; therefore, it did not exhibit sufficient efficacy [9, 10]. In addition, in the circulation, all NPs could induce diuresis and hypotension [5]. If CNP(1–22) was administered at high doses, it might cause a decrease in systemic vascular resistance and blood pressure in patients [11]. Therefore, the cardiovascular side effects associated with the use of CNP as a therapeutic agent could never be ignored. It was reported that exogenous CNP(1–22) improved endochondral ossification and accelerated bone growth in mice after constant intravenous infusion at a large dose only [12]. These findings indicate the difficulty of the commercial clinical applications of CNP.

In a previous study, we showed that the C-terminal part of ghrelin played an important role in the pharmacokinetic (PK) profile and growth hormone-releasing activity of ghrelin [13]. Furthermore, this finding could be applicable to the other peptides, such as motilin and CNP [14, 15]. The application of C-terminal part of ghrelin resulted in the higher stability of CNP analogs, compared to that of the native form; it also improved their bioactivity as stimulators of endochondral bone growth.

In this study, we indicated that optimization of the peptide sequence and the dosage regimen were key factors for successful therapeutic drug development using the CNP/NPR-B signaling pathway. Then, we used ASB20123 as a novel CNP derivative to test our hypothesis. This might be a novel pharmacological approach based on the biology and chemistry of CNP with a unique perspective in peptide drug development.

## Materials and methods

### Peptides

Alpha-type human ANP (α-hANP), CNP(1–22), and CNP analogs were produced from *Escherichia coli* using recombinant DNA technology. Human ghrelin was synthesized by chemical condensation of the N-terminal 7 amino acid peptide and the recombinant 21-residue C-terminal fragment, as previously reported [16].

**Fig 1:**
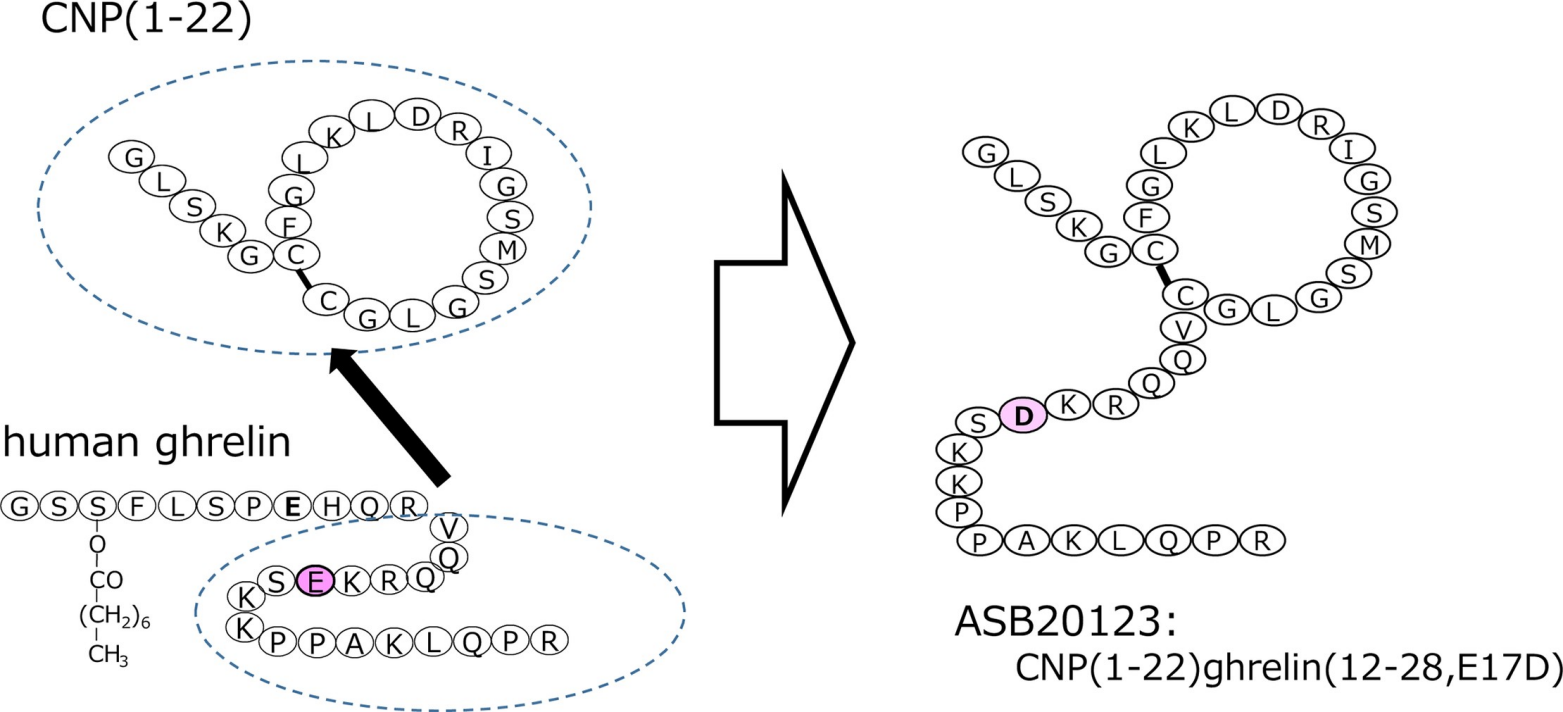
The amino acid sequences and structures of CNP(1–22), human ghrelin, and ASB20123. CNP(1–22) is a 22-amino acid (AA) natriuretic peptide. ASB20123 is a 39-AA-designed chimeric natriuretic peptide composed of CNP(1–22) and the 17-AA C-terminal fragment of human ghrelin(12–28). It has a single amino acid exchange as CNP(1–22)ghrelin(12–28, E17D).

We have previously prepared several CNP/ghrelin chimeric peptides and evaluated NPR-B receptor agonist activity *in vitro* and pharmacokinetic and pharmacodynamics profiles *in vivo* [15]. Based on these results, we designed a novel CNP/ghrelin chimeric peptide, ASB20123. ASB20123 is a CNP/ghrelin chimeric peptide with exchange of a single amino acid of CNP(1–22)/ghrelin(12–28). The molecular weight of ASB20123 is 4183.9, which is approximately twice as large as that of CNP(1–22) (2197.6). We produced ASB20123 from recombinant DNA inserted in *E*. *coli*., and the productivity of ASB20123 was higher than that of CNP(1–22)/ghrelin(12–28). Amino acid sequences of CNP(1–22), human ghrelin, and ASB20123 are shown in Fig 1. They were verified by electrospray ionization mass spectrometry and amino acid composition analysis. All peptides used in this study were purified by high-performance liquid chromatography, and the purity of each peptide was > 95%.

### Animals

Sprague Dawley (SD) rats and ICR mice (Charles River Laboratories, Japan) were used in the current study. The animals were housed in a humidity- and temperature-controlled environment with an automatic 12-h light/dark cycle. They were provided with a standard, pelleted lab chow diet (CRF-1, Oriental Yeast Co., Ltd., Japan) and tap water *ad libitum*. All animal experiments were conducted in accordance with the Guidelines for Animal Experiments of Asubio Pharma Co., Ltd., and were approved by the Animal Research Committee of Asubio Pharma Co., Ltd (Permit number: AE080108, AEK-15-086, AEK-15-056R, and AEK-15-127).

### *In vitro* functional assays to assess receptor specificity

To evaluate the human NPR (hNPR) agonist activities of the test compounds, we used Chinese hamster ovarian (CHO) cells stably expressing hNPR-A or hNPR-B [15, 17]. Each compound was added to the cells in duplicate and incubated for 15 min. Cells were lysed, and cyclic guanosine monophosphate (cGMP) concentration in each sample was determined by competitive enzyme-linked immunosorbent assay (ELISA) using the CatchPoint cGMP fluorescent assay kit (Molecular Devices Corporation, USA) and FlexStation (Molecular Devices Corporation, USA), according to the manufacturer’s instructions. To evaluate growth hormone secretagogue receptor 1a (GHS-R1a) agonist activities, we used CHO cells stably expressing rat GHS-R1a [16, 18]. Changes in intracellular Ca^2+^ concentrations ([Ca^2+^]_i_) were measured using FRIPR calcium 3 assay kit (Molecular Devices Corporation, USA) and FlexStation.

### PK studies in rats

Seven or 8-week-old male SD rats were used. As the anesthesia method in the first part of the study period, rats were anesthetized with intraperitoneal injection of 50 mg/kg sodium pentobarbital (NEMBUTAL Sodium Solution, Sumitomo Dainippon Pharma Co., Ltd., Japan). However, according to the recent guidelines, we changed the anesthetic to Inactin hydrate (Sigma-Aldrich Co. LLC., USA) at 100 mg/kg during the remainder of the study period. A cannula containing 100 U/mL heparin sodium in physiological saline was inserted into the femoral artery. Rats received CNP(1–22) or ASB20123 by intravenous (*iv*) injection into the tail vein or subcutaneous (*sc*) injection into the back at a dose volume of 1 mL/kg. After the designated period, a small amount of blood was collected via the cannula into a test tube containing aprotinin (Bovine, Nacalai Tesque, Inc., Japan) and ethylenediaminetetraacetic acid (EDTA-2Na, Wako Pure Chemical Industries, Ltd., Japan). The collected blood was centrifuged at 14,000 ×*g*, 4°C, and plasma was collected. CNP immunoreactivity in the plasma was determined by radioimmunoassay (RIA) using antiserum against the 17-membered internal disulfide ring of CNP and [^125^I]-labeled [Tyr^0^]-CNP(1–22) as a tracer [15]. The plasma concentration of cGMP was determined by RIA using the Yamasa cGMP assay kit (YAMASA Corporation, Japan).

### *In vivo* evaluation of cGMP production in auricular cartilage of mice

Six-week-old ICR male mice (*n* = 4 or 5/group) received a *sc* bolus injection of CNP(1–22) at 1600 nmol/kg, ASB20123 at 200 nmol/kg, or vehicle solution. After the designated period, the animals were anesthetized with isoflurane (Mylan Inc., UK), and blood was collected from the inferior *vena cava* into a test tube containing EDTA-2Na and centrifuged to harvest plasma. A portion of ear auricle was isolated and boiled in hot water to inactivate cGMP-degrading enzymes. The auricular cartilage samples were trimmed, separated from other tissue, and homogenized in 6% perchloric acid solution (Wako Pure Chemical Industries, Ltd., Japan). The samples were centrifuged, and aliquots of the supernatants were collected and neutralized with KOH solution. cGMP concentrations in all samples were measured using commercially available EIA kits (Amersham cGMP enzyme immunoassay Biotrak (EIA) System, GE Healthcare Company, USA).

### *In vivo* pharmacological studies in mice and rats

Three-week-old juvenile female ICR mice (*n* = 5 or 10/group) received ASB20123 *sc* bolus injections once daily for 8 weeks. Seven-week-old male SD rats (*n* = 5/group) received ASB20123 as multiple *sc* bolus injections or a continuous *sc* infusion using an osmotic mini-pump (ALZET osmotic pump, Durect Corporation, USA) for 1 or 12 weeks. Growth rate of each animal was monitored throughout the treatment and washout periods. Body weight, body (naso-anal) length, and tail length were measured weekly, and femoral and tibial lengths were measured with digital calipers on the final day. Osteocalcin concentration in mouse serum was measured as a marker of bone formation using commercially available ELISA kits (Mouse Osteocalcin EIA kit, Biomedical Technologies Inc., USA). Anti-ASB20123 antibody in mouse serum was measured using commercially available ELISA kits (Protein detector ELISA Kit, Kirkegaard & Perry Laboratories, Inc., USA) and ASB20123. The blood samples were collected from the posterior vena cava at the end of the dosing period and centrifuged to obtain serum samples. CNP immunoreactivity in rat plasma was determined by RIA as mentioned in the method of PK studies. The blood samples were collected from cervical vein at 15 min after *sc* bolus dosing or before necropsy for animals given a continuous *sc* infusion.

### Histological analysis of growth plates in rats

The femur and tibia were dissected from the rats, fixed in 10% neutral buffered formalin, and decalcified in a mixture of 10% formic acid and formalin solution for a week. The samples were embedded in paraffin, sectioned, stained with hematoxylin and eosin (HE), and examined microscopically. The thickness of growth plates at the proximal and distal ends of the femur and tibia, respectively, was measured under a light microscope. The growth plate was measured at nine sites for the proximal end of the femur and five sites for the distal end of the tibia. The average of these values was considered the growth plate thickness for each bone of each rat. In addition, the growth plate thickness of the other end of the femur (distal) or tibia (proximal) was measured at one site of the central of the growth plate.

### Statistical analysis

Statistical analysis of the data was performed using two-way factorial analysis of variance (ANOVA) followed by Dunnett's test as a *post-hoc* test. *P*-values < 0.05 were considered statistically significant. Data are expressed as the mean ± standard deviation (SD).

## Results

### Receptor agonist activity of ASB20123

**Fig 2:**
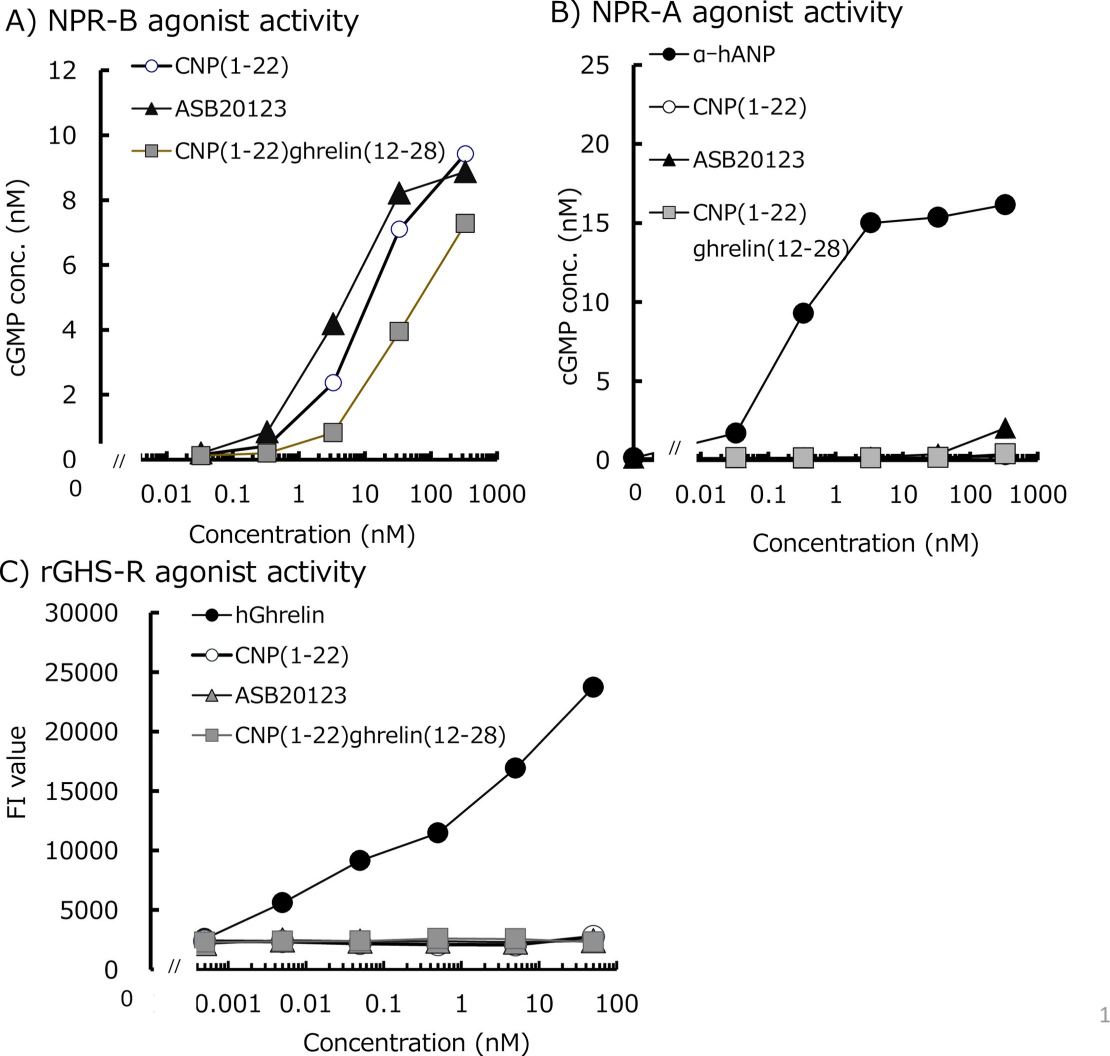
Receptor agonist activity of CNP and its derivatives. (A) NPR-B agonist activity of CNP(1–22), CNP(1–22)/ghrelin(12–28), and ASB20123 was evaluated based on cGMP production in CHO cells stably expressing human NPR-B. (B) NPR-A agonist activity of α-hANP, CNP(1–22), CNP(1–22)/ghrelin(12–28), and ASB20123 was evaluated based on cGMP production in CHO cells stably expressing hNPR-A. (C) GHS-R agonist activity of human ghrelin, CNP(1–22), CNP(1–22)/ghrelin(12–28), and ASB20123 based on the changes in [Ca^2+^]_i_ in CHO cells stably expressing rat GHS-R1a. Each value represents the mean of duplicate assays.

We evaluated the potency and specificity of ASB20123 using cell-based receptor agonist assays. Fig 2A and 2B show the cGMP-producing activity of each peptide in CHO cells stably expressing human NPR-B and NPR-A. CNP(1–22), CNP(1–22)/ghrelin(12–28), and ASB20123 increased cGMP production in CHO cells expressing human NPR-B in a concentration-dependent manner. There was little difference in NPR-B agonist activity between ASB20123 and the endogenous ligand, CNP(1–22). In contrast, CNP(1–22) and its derivatives were not agonists of NPR-A. These peptides did not increase [Ca^2+^]_i_ in CHO cells expressing rat GHS-R1a (Fig 2C).

### PK profile in rats

**Fig 3:**
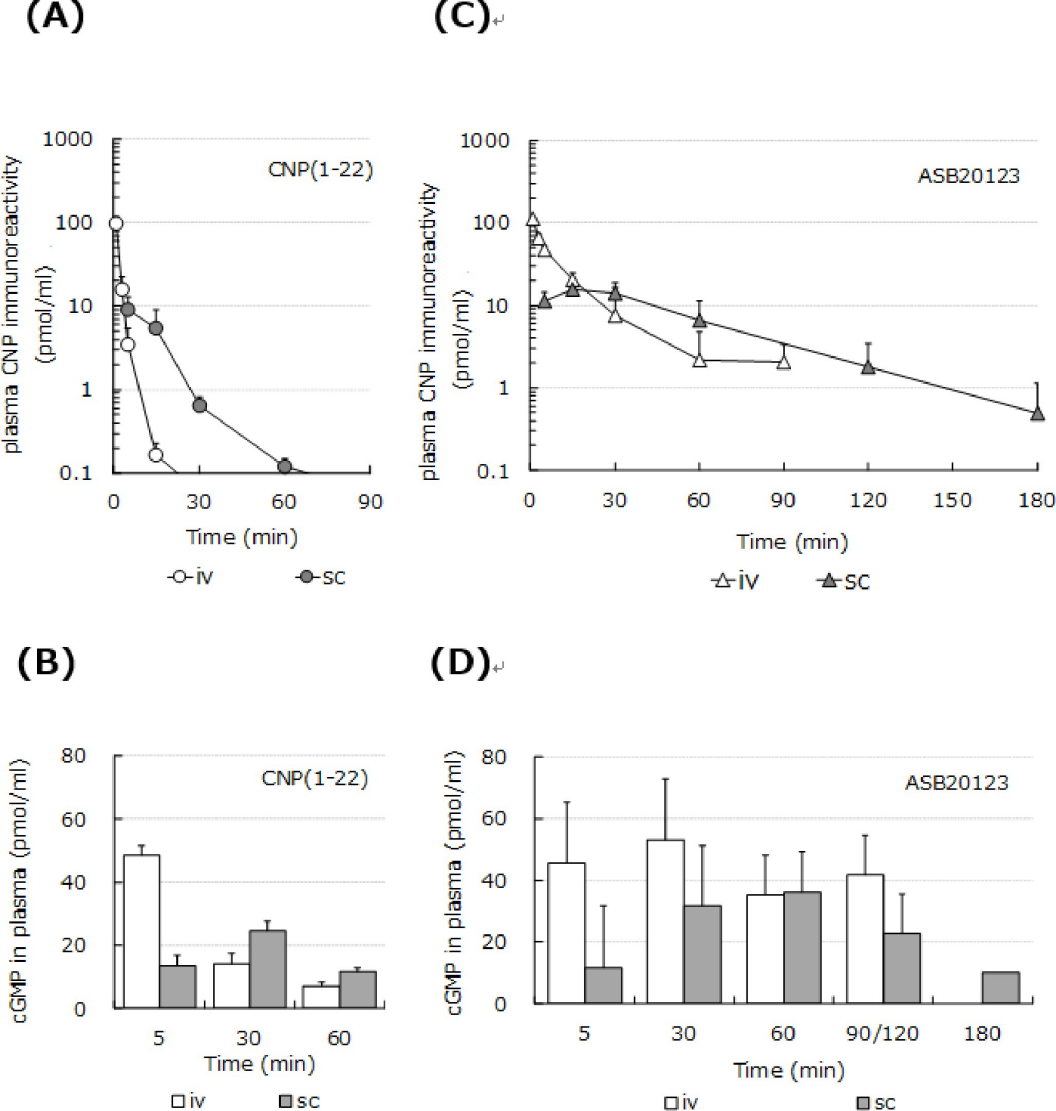
PK/pharmacodynamic profiles of CNP(1–22) and ASB20123 in rats. Plasma CNP immunoreactivity-time curves (the upper panels) and cGMP concentrations (the lower panels) after a single *iv* (20 nmol/kg) or *sc* (50 nmol/kg) dose of CNP(1–22) (A, B) or ASB20123 (C, D) in anesthetized rats. Each value represents the mean ± SD of 3 rats.

Plasma concentration-time curves for CNP immunoreactivity after single *iv* (20 nmol/kg) or *sc* (50 nmol/kg) bolus injection of CNP(1–22) or ASB20123 in rats are depicted in Fig 3A and 3C. After *iv* or *sc* dosing, the area under the curve (AUC) after administration of ASB20123 was 4- or 6.8 fold higher than that of CNP(1–22) (Table 1 and Table 2). These data indicated that ASB20123 was resistant to proteolytic inactivation with slow clearance from the circulation. In addition, the distribution volume at steady state (Vdss) of ASB20123 was 6.7-fold larger than that of CNP(1–22) after *iv* dosing (Table 1). In addition, plasma cGMP concentration after ASB20123 dosing was higher than that after CNP(1–22) dosing at almost all times, and it was maintained at a higher level than the basal level for 180 min after *sc* dosing (Fig 3B and 3D). These data indicated that ASB20123 could be transported across the vascular wall and reach NPR-B in peripheral tissues more easily than CNP(1–22).

**Table 1:** PK parameters of CNP immunoreactivity after a single intravenous administration of CNP(1–22) or ASB20123 in rats.

Peptides	Dose	AUC_0→∞_		MRT_0→∞_		T_1/2_		CL_tot_		Vd_ss_	
	(nmol/kg)	(pmol・min/mL)		(min)		(min)		(mL/min/kg)		(mL/kg)	
CNP(1–22)	20	320	± 54	1.02	± 0.18	1.42	± 0.45	63.9	± 11.9	64.2	± 5.1
ASB20123		1281	± 502	24.1	± 2.6	31.3	± 4.8	17.9	± 8.9	429	± 204

PK parameters were calculated from the individual plasma CNP immunoreactivity-time curves after a single intravenous administration of each peptide in anesthetized rats. Each value represents the mean ± SD of 3 rats. MRT = mean residence time, CL_tot_ = total clearance, T_1/2_ = half-life period, BA = bioavailability.

**Table 2:** PK parameters of CNP immunoreactivity after a single subcutaneous administration of CNP(1–22) or ASB20123 in rats.

Peptides	Dose	Cmax		Tmax		AUC_0→∞_		MRT_0→∞_		T_1/2_		BA	
	(nmol/kg)	(pmol/mL)		(min)		(pmol・min/mL)		(min)		(min)		(%)	
CNP(1–22)	50	9.02	± 3.74	5.0	± 0.0	152	± 73	13.9	± 3.4	10.0	± 5.0	19	± 9
ASB20123		15.60	± 0.95	20.0	± 8.7	1037	± 96	49.6	± 6.0	32.1	± 8.0	32	± 3

PK parameters were calculated from the individual plasma CNP immunoreactivity-time curves after a single subcutaneous administration of each peptide in anesthetized rats. Each value represents the mean ± SD of 3 rats. MRT = mean residence time, CL_tot_ = total clearance, T_1/2_ = half-life period, BA = bioavailability.

### Structure-function relationships in the efficiency of distribution to auricular cartilage in mice

**Fig 4:**
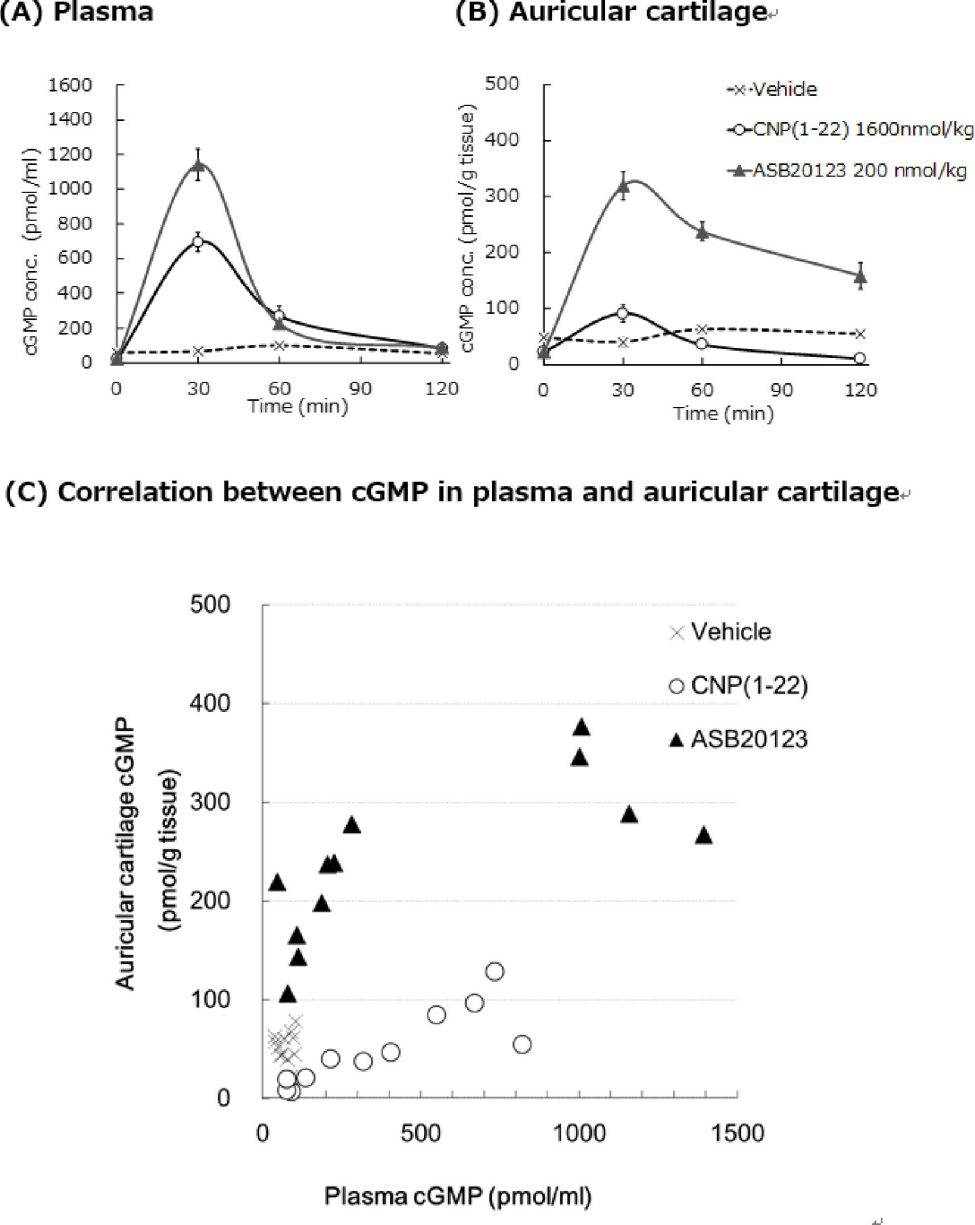
cGMP concentration-time curves in plasma and auricular cartilage after a single *sc* dose of CNP(1–22) or ASB20123 in mice. Plasma (A) or auricular cartilage (B) cGMP concentration-time curves after a single *sc* dose of CNP(1–22) at 1600 nmol/kg or ASB20123 at 200 nmol/kg in mice. Correlation between cGMP concentrations in plasma and in auricular cartilage at each sampling point (C). Each value represents the mean ± SD of 4 mice (A, B) or individual value (C).

For estimation of the efficiency of the distribution of each peptide from plasma to cartilage, we compared cGMP concentrations in the plasma and auricular cartilage after a single *sc* bolus injection of CNP(1–22) at 1600 nmol/kg or ASB20123 at 200 nmol/kg to mice. Data are shown in Fig 4. After ASB20123 dosing, cGMP concentrations in the plasma (Fig 4A) and auricular cartilage (Fig 4B) were clearly higher than those after CNP(1–22) dosing. In particular, cGMP concentration was still higher in the auricular cartilage at 120 min after ASB20123 dosing than that in the vehicle-treated control group. The correlation between cGMP concentration in the auricular cartilage and that in plasma after ASB20123 dosing is shown in Fig 4C. These results show that ASB20123 efficiently increased cGMP concentration in auricular cartilage. ASB20123 was more easily distributed to the target cartilage tissue with higher concentration and remained for a longer time than CNP in its native form.

### Effects of ASB20123 on skeletal growth in juvenile mice

**Fig 5:**
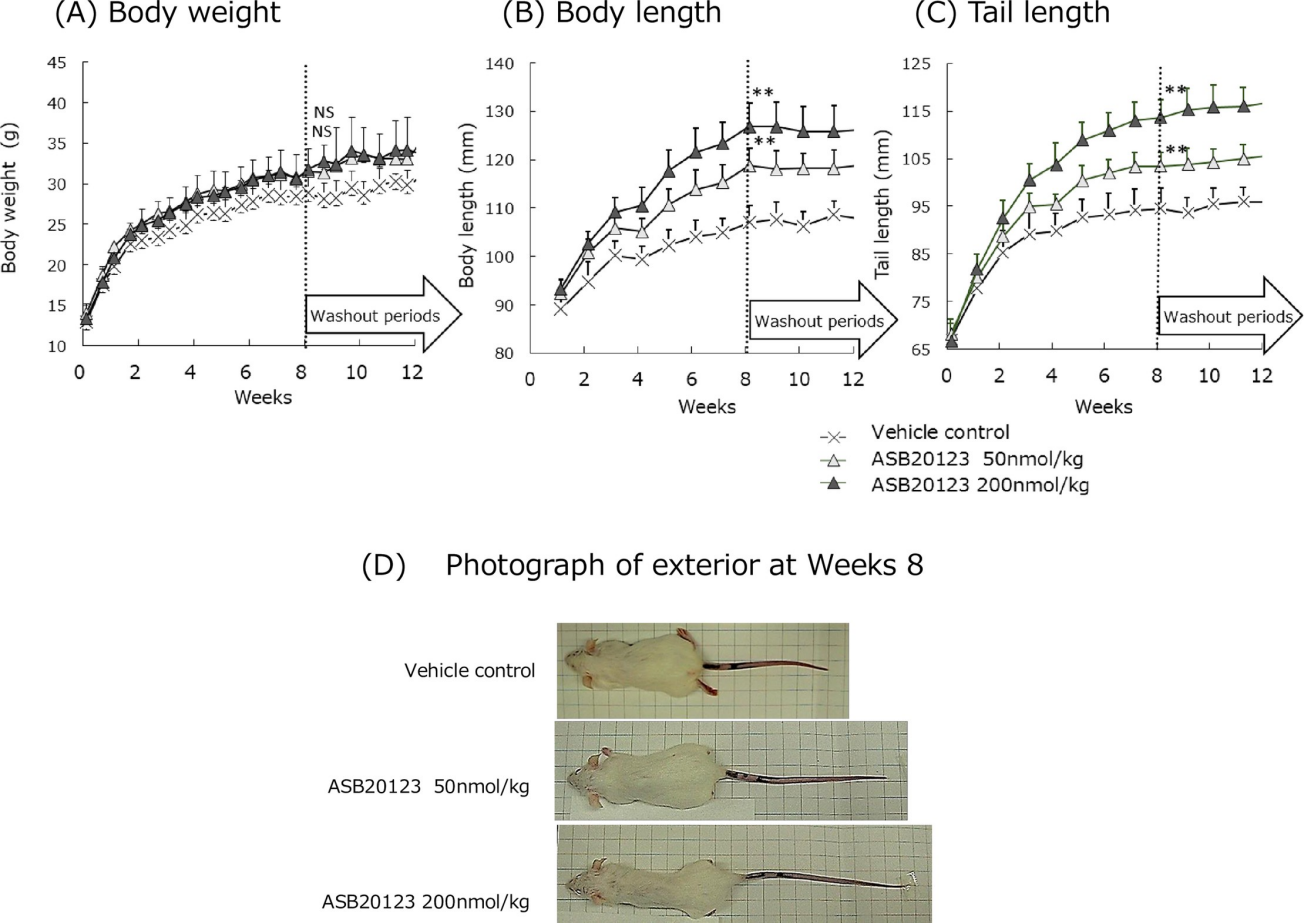
Growth curves of female juvenile ICR mice treated with ASB20123 *sc* during the 8 weeks of the dosing period and the 4 weeks of the washout period. Body weight (A), body length (B) and tail length (C) data are shown in the upper panels, and the photographs in the lower panel represent the gross appearance of mice at Day 56 (D). Each value represents the mean ± SD of 10 (for the dosing period) or 5 mice (for the washout period). NS: not significant (*p* > 0.05), *: significant difference (*p* < 0.05) compared to the control group using Dunnett’s test.

The *in vivo* pharmacological activities of ASB20123 were evaluated in juvenile female ICR mice. Data are shown in Fig 5. ASB20123 was administered *sc* to 3-week-old female mice (*n* = 10/group) for 8 weeks at doses of 0 (vehicle control), 50, and 200 nmol/kg/day. Body length and tail length of the mice in the ASB20123-treated groups were longer than those in the vehicle-treated control group (Fig 5D). Skeletal growth-accelerating activities of ASB20123 were dose-dependent and statistically significant (Fig 5B and 5C). The serum concentration of osteocalcin, a bone formation marker, was significantly greater in the high dose group compared to the control group at the end of the dosing period (Table 3). Moreover, the effects of ASB20123 on body weight were minimal and not significantly different among groups (Fig 5A). There was no significant difference in the growth rates among groups during the 4-week washout period (Fig 5B and 5C). It was indicated that CNP-induced skeletal growth acceleration quickly disappeared upon drug withdrawal. In addition, anti-ASB20123 antibody was not detected in all mice received ASB20123 for 8 weeks, and after 4-week washout period (S1 Table).

**Table 3:** Serum osteocalcin concentration of juvenile female ICR mice with multiple *sc* bolus injections of ASB20123 for 8 weeks.

Peptides	Dose (nmol/kg/day)	Serum osteocalcin conc. (ng/mL)	
ASB20123	0 (vehicle)	27.2	± 8.2
	50	33.5	± 6.9 ^NS^
	200	47.5	±13.2 **

Each value represents the mean ± SD of 5 mice. NS: not significant (*p* > 0.05)

**: *p* < 0.01 compared to the control group using Dunnett’s test.

These results indicated that the pharmacological effects of ASB20123 were specific to long bone elongation *via* stimulation of endochondral ossification.

### Effects of continuous *sc* infusion of ASB20123 in rats

**Fig 6:**
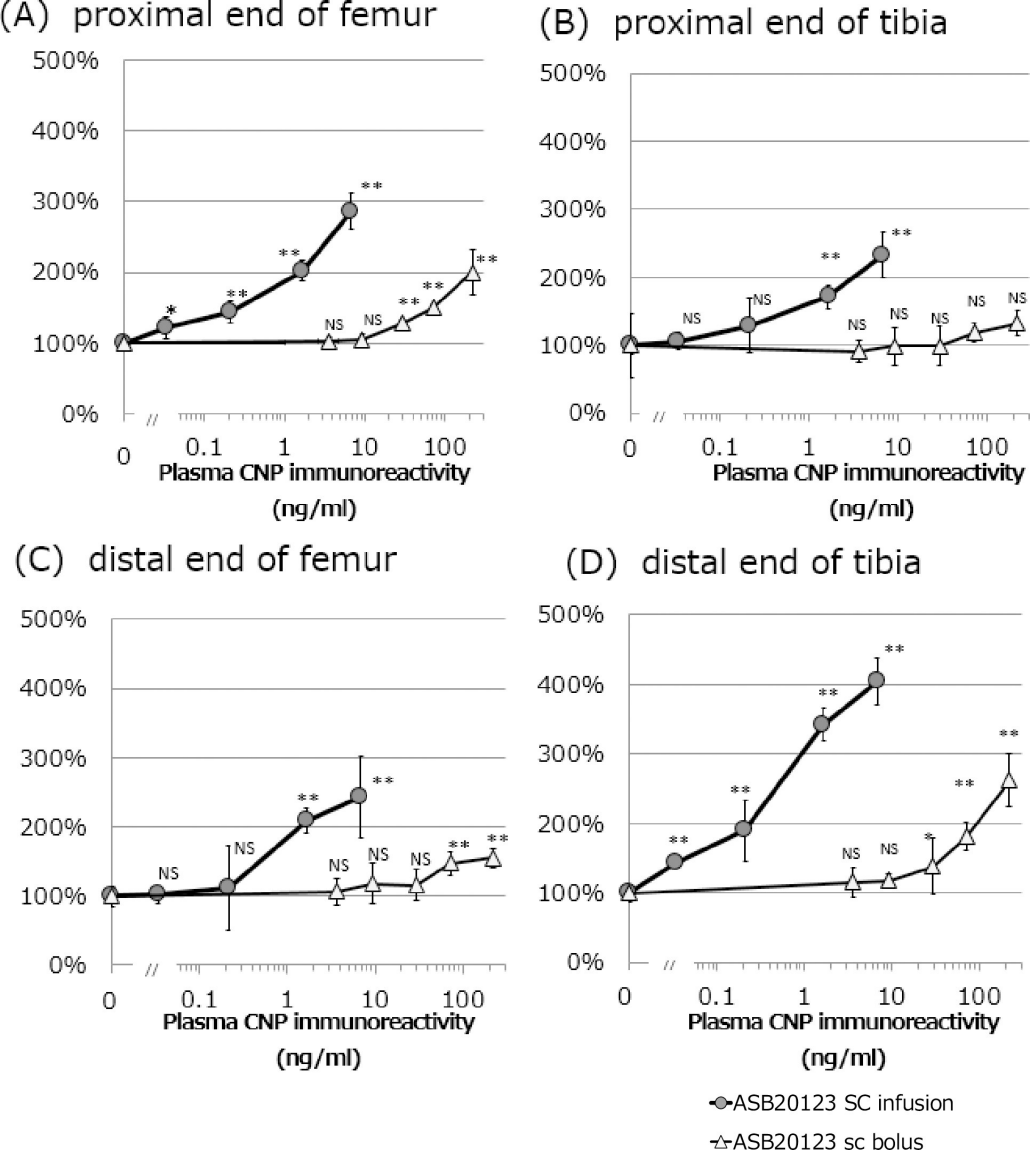
**Correlation between plasma CNP immunoreactivity and growth plate thickness of lower limbs [proximal ends of femur (A) and tibia (B), and distal ends of femur (C) and tibia (D)] in rats after *sc* bolus injections or infusion of ASB20123 at 0, 0.005, 0.015, 0.05, 0.15, and 0.5 mg/kg/day for 7 days.** Each value represents the mean ± SD of 4 or 5 rats. The horizontal axis indicates the mean plasma CNP immunoreactivity concentration at 15 min after the 1^st^ dose for the *sc* bolus injection groups or mean plasma CNP immunoreactivity on the 2^nd^ day for the *sc* infusion groups. The vertical axis indicates the mean percentage of growth plate thickness compared to the respective control groups. Plasma CNP immunoreactivity after *sc* infusion of ASB20123 at 0.005 mg/kg/day was not detected. NS: not significant (*p* > 0.05), *: *p* < 0.05, **: *p* < 0.01 compared to the control group (0 mg/kg/day) using Dunnett’s test (logarithm conversion).

We also analyzed whether continuous *sc* infusion of ASB20123 to rats could accelerate skeletal growth, compared to the effects of multiple *sc* bolus injections. ASB20123 was administered to 7-week-old male rats at 0, 0.005, 0.015, 0.05, 0.15, and 0.5 mg/kg/day (1.2, 3.6, 12, 36 and 120 nmol/kg/day) for 7 days, and body and tail lengths were measured (Table 4 and Table 5). Continuous *sc* infusion of ASB20123 at 0.15 and 0.5 mg/kg/day for only 7 days resulted in a dose-dependent increase in the growth rate (body length), whereas multiple *sc* bolus injections showed no effects at these same doses. The femoral and tibial growth plate sizes observed at the end of dosing are shown in Fig 6. The mean growth plate thickness increased dose-dependently under both dosing conditions; however, *sc* infusion was more effective at the same plasma concentration (C_ss_ or C_max_).

**Table 4:** Body measurements and growth rate in male rats receiving multiple subcutaneous bolus injections of ASB20123 for 7 days.

Dose (mg/kg/day)	Weight or length on the final day			Growth rate		
	Body weight	Body length	Tail length	Body weight	Body length	Tail length
	(g)	(mm)	(mm)	(g/week)	(mm/week)	(mm/week)
0 (vehicle)	317 ± 11	212.4 ± 6.4 ^NS^	201.6 ± 6.4 ^NS^	56 ± 3 ^NS^	12.6 ± 5.7 ^NS^	14.2 ± 2.6 ^NS^
0.005	315 ± 21 ^NS^	209.6 ± 3.2 ^NS^	197.0 ± 3.9 ^NS^	49 ± 10 ^NS^	13.6 ± 3.4 ^NS^	11.4 ± 4.0 ^NS^
0.015	315 ± 15 ^NS^	212.8 ± 3.8 ^Ns^	208.0 ± 7.2 ^NS^	50 ± 9 ^NS^	11.6 ± 2.1 ^NS^	12.4 ± 3.8 ^NS^
0.05	317 ± 15 ^NS^	211.8 ± 4.0 ^NS^	205.2 ± 5.6 ^NS^	53 ± 6 ^NS^	11.4 ± 2.9 ^NS^	14.2 ± 4.3 ^NS^
0.15	322 ± 11 ^NS^	215.4 ± 2.4 ^NS^	201.4 ± 5.7 ^NS^	58 ± 6 ^NS^	14.8 ± 3.7 ^NS^	9.6 ± 3.5 ^NS^
0.5	316 ± 10 ^NS^	210.4 ± 2.6 ^NS^	206.8 ± 5.4 ^NS^	48 ± 4 ^NS^	11.6 ± 4.2 ^NS^	12.0 ± 2.7 ^NS^

Each value represents the mean ± SD of 4 or 5 rats. NS: not significant (*p* > 0.05) compared to the control group using Dunnett’s test.

**Table 5:** Body measurements and growth rate in male rats receiving continuous subcutaneous infusion of ASB20123 for 7 days.

Dose (mg/kg/day)	Weight or length on the final day			Growth rate		
	Body weight	Body length	Tail length	Body weight	Body length	Tail length
	(g)	(mm)	(mm)	(g/week)	(mm/week)	(mm/week)
0 (vehicle)	333 ± 13 ^NS^	215.0 ± 3.5 ^NS^	205.4 ± 4.2 ^NS^	53 ± 7 ^NS^	8.4 ± 3.4 ^NS^	5.4 ± 3.6 ^NS^
0.005	337 ± 14 ^NS^	221.0 ± 3.3 ^NS^	211.6 ± 4.2 ^NS^	53 ± 7 ^NS^	13.2 ± 7.0 ^NS^	6.4 ± 2.3 ^NS^
0.015	328 ± 14 ^NS^	219.6 ± 3.4 ^NS^	207.2 ± 2.3 ^NS^	48 ± 7 ^NS^	11.0 ± 2.0 ^NS^	5.4 ± 1.9 ^NS^
0.05	329 ± 12 ^NS^	219.8 ± 2.4 ^NS^	208.8 ± 4.6 ^NS^	49 ± 8 ^NS^	14.6 ± 3.5 ^NS^	6.2 ± 3.3 ^NS^
0.15	343 ± 4 ^NS^	222.8 ± 4.5*	210.3 ± 6.1 ^NS^	59 ± 3 ^NS^	16.5 ± 4.4*	10.0 ± 2.0 ^NS^
0.5	330 ± 22 ^NS^	226.4 ± 4.6**	215.6 ± 1.8**	48 ± 20 ^NS^	22.2 ± 4.0**	11.0 ± 4.7*

Each value represents the mean ± SD of 4 or 5 rats. NS: not significant (*p* > 0.05)

*: *p* < 0.05

**: *p* < 0.01 compared to the control group using Dunnett’s test.

### Skeletal growth-accelerating effects of long-term ASB20123 dosing in rats

**Fig 7:**
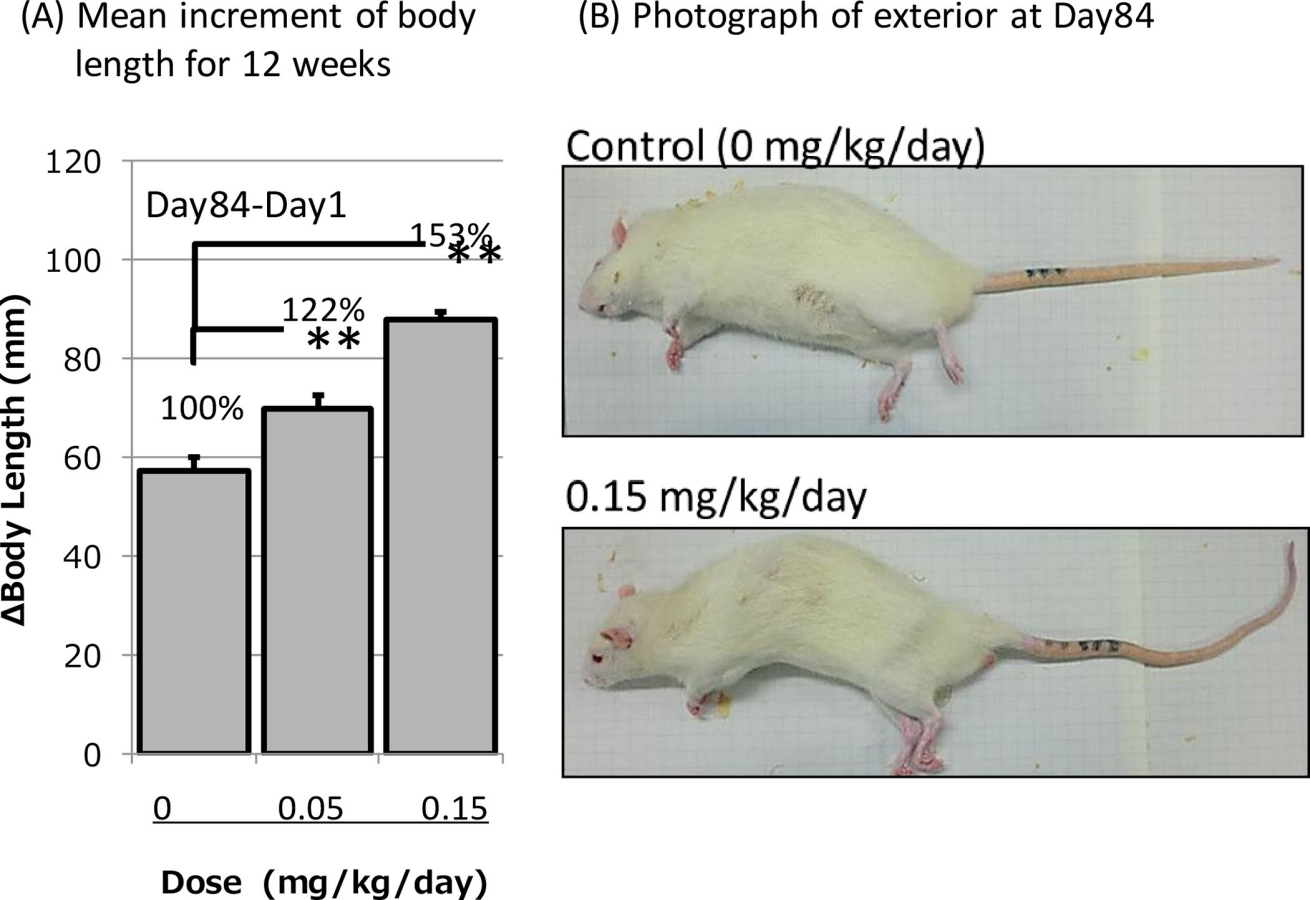
Increase in body length of male rats receiving *sc* infusion of ASB20123 at 0.05 and 0.15 mg/kg/day for 12 weeks. Each value represents the mean ± SD of 4 or 5 rats, **: significant (*p* < 0.01) compared to the control group using Dunnett’s test. (A) Mean increase in body length after 12 weeks (84 days). (B) Photographs of the exterior appearance on day 84.

A long-term study using ASB20123 *sc* infusion at pharmacologically effective doses was conducted in 7-week-old male SD rats. ASB20123 was infused *sc* to rats (*n* = 5/group) using an osmotic pump at doses of 0 (vehicle control), 0.05, and 0.15 mg/kg/day (12 and 36 nmol/kg/day) for 12 weeks. The mean increase in body length during the dosing period is shown in Fig 7A. The increase in body length at the final day was significantly different and dose-dependently. The growth rates in the vehicle-treated control, 0.05 and 0.15 mg/kg/day ASB20123-treated groups were 57.2 ± 3.6, 70.0 ± 5.0 and 87.8 ± 6.2 mm/12 weeks, respectively. In particular, rats in the 0.15 mg/kg/day ASB20123-treated group showed an increase in the curvature of the spine and apparent abnormal curvature in the tail (Fig 7B). These phenotypes might be attributable to overgrowth, and the doses used in this study might be considered as excessive dose in pharmacological studies using normal adolescent rats.

#### Discussion

In the previous study, we designed and evaluated some CNP/ghrelin chimeric peptides as novel CNP derivatives [15]. One of the resultant peptides, CNP(6–22)/ghrelin(12–28) displayed improved biokinetics, compared to CNP(1–22), and repeated *sc* administration of CNP(6–22)/ghrelin(12–28) to mice resulted in significant acceleration of longitudinal bone growth, whereas CNP(1–22) treatment showed no effect on skeletal growth under the same conditions. In the current study, we prepared another novel CNP/ghrelin chimeric peptide ASB20123 and evaluated its pharmacological activities. ASB20123 is composed of the full-length 22-amino acid human CNP(1–22) and the C-terminus region of human ghrelin with a single amino acid substitution (CNP(1–22)/ghrelin(12–28, E17D)). ASB20123 had full NPR-B agonist activity and clearly increased AUC compared to CNP(1–22) [Table 1, Fig 2]. In this study, we also showed that multiple *sc* bolus injections of ASB20123 potently stimulated bone growth in a dose-dependent manner. In addition, *sc* infusion was more effective than multiple *sc* bolus injections. We consider that the effects of CNP/NPR-B signaling using normal rats are likely to be reflected in the dwarf models, because BMN-111, a CNP analog, treatment increased the body length in *Fgfr3*^*ach*^ mice [19] and in a Crouzon syndrome mouse model [20].

ASB20123 *sc* infusion was able to significantly accelerate skeletal growth after a single week of infusion. It was reported that transgenic mice in which CNP is expressed in the liver with mild increase of plasma CNP. These mice exhibited skeletal overgrowth phenotype without a change in systolic blood pressure, whereas mice with high overexpression of CNP showed a significant reduction of systolic blood pressure, compared to that in wild-type littermates [21]. These results indicated that CNP exhibited hypotensive effects. Although the hypotensive effects of CNP are weaker than those of other NPs, such as ANP and BNP, its use is associated with the risk of hypotension [22]. In fact, clinically significant hypotension has been reported with other CNP derivatives and might be a dose-limiting factor in clinical practice [23, 24].

Long-term use of CNP for treatment of growth failure in pediatric patients might increase the risk of unpredicted adverse events, which could also be affected by inappropriate dosage and the physical condition of the patients [24]. Peptides should have wide safety margins for home use in the outpatient setting in the clinical setting; therefore, we suggested that continuous *sc* infusion might be one of the superior methods to promote bone growth. This kind of formulation might allow the maintenance of a safe and effective plasma concentration, even when continuously administered for a long period. We showed that *sc* infusion dosage allowing chronic exposure to ASB20123 might be suitable as skeletal growth-accelerating agents. This might be a good strategy to avoid their on-target toxicological effects, such as hypotension.

The growth plate thickness increased significantly after continuous *sc* infusion at the dosage of 0.05 mg/kg/day of ASB20123 for 7 days, but the body length was not significantly affected. Meanwhile, the body length increased significantly at the same dosage for 12 weeks, and rats given the higher dosage of 0.15 mg/kg/day showed overgrowth. These results indicate that long-term administration results in more effective bone elongation at lower dosage, and it would be necessary to set the suitable dosage for each patient. Our results suggest that the thickness of the growth plate could be a good marker to evaluate the effective dosage in the clinic, because it can be monitored using radiographic examination, computed tomography and magnetic resonance imaging in humans [25, 26].

The growth plate is the cartilage area of growing structures near the ends of the long bones. Cartilages, such as growth plate, do not have blood vessels, nerves, or lymphatics, and their function is dependent on the molecular composition of the extracellular matrix (ECM) [27]. The ECM consists mainly of proteoglycan, hyaluronic acid (HA), and collagen [28]. In this study, cGMP concentration in mouse auricular cartilage after a single *sc* dose of ASB20123 was higher than that after CNP(1–22) dosing. ASB20123 had a superior affinity to cartilages as a target structure. The C-terminal part of ghrelin might change the *in vivo* PK of ASB20123 to allow targeted delivery to cartilage structures, including the growth plate.

The C-terminal part of the ghrelin includes a BX7B motif, where “B” refers to a basic amino acid (arginine or lysine), and “X” represents any non-acidic amino acid [29]. It has been reported that clusters of basic amino acids, such as BX7B motifs, are implicated in the interaction between HA molecules. In particular, HA has a pronounced hydrophilic capacity and can form viscoelastic meshworks that can facilitate cell proliferation and migration *via* interaction with specific cell surface receptors or binding proteins to directly regulate cell behavior [30, 31].

These basic amino acid clusters might explain the difference between CNP(1–22) and ASB20123 (S1 Fig). We prepared CNP(1–22) and ASB20123 dissolved in vehicles containing ECM and delivered them to rats as *sc* bolus injections at 0.58 mg/kg for evaluation of their *in vivo* effects (S2 Fig). When ASB20123 was dissolved in 1% fully synthetic HA solution (ARTZ Intra-articular Injection 25 mg, Kaken Pharmaceutical Co., Ltd.) or 10% sulfated glycosaminoglycan solution (extracted from bovine tracheal cartilage, Adequan, Luitpold Pharmaceuticals), the time to reach the maximum plasma CNP concentration (Cmax) was delayed, and the AUC was higher than that when ASB20123 was dissolved in saline. These results mean that the bioavailability was improved, and absorption from the site of administration was delayed and maintained for a long time. In other words, the stability and retention of ASB20123 was improved in the presence of ECM.

Interstitial diffusion of macromolecules, such as HA and glycosaminoglycan, is affected by their physiochemical characteristics, including their size, charge, and hydrophilicity, as well as their interactions with the endogenous components present in the interstitium [32]. Interaction between ASB20123 and the endogenous molecules might play a role in the diffusion and absorption processes. In contrast, the PK profile of CNP(1–22) was not affected by its vehicle components. The C-terminal part of ASB20123 might interact with ECM components, such as HA and glycosaminoglycan, resulting in a delay of its absorption from the *sc* tissue to the circulation and improvement of its stability. Interestingly, the N-terminal part of CNP(1–53) also includes a BX7B motif similarly to the C-terminal part of ASB20123 (S1 Fig). In fact, we showed that exogenous administration of CNP(1–53) induced skeletal growth in mice more potently than that induced by CNP(1–22) administration (S3 Fig).

Pro-CNP is processed to generate 22-residue (CNP(1–22)) and 53-residue (CNP(1–53)) peptides. CNP, the endogenous ligand of NPR-B, is present mainly as CNP(1–53) in various tissues, including the brain [33], cultured human aortic endothelial cells [34], and rat kidneys [35]. CNP(1–53) is resistant to proteolytic inactivation by neutral endopeptidase (NEP); however, it can hardly be detected in the plasma. In contrast, the molecular form of CNP(1–22) has been identified in human plasma at very low concentrations. Therefore, CNP(1–53) might act as an autocrine/paracrine regulator of endochondral ossification. One of the attractive features of ASB20123 as a novel drug candidate is the addition of CNP(1–22), using the structural basis of the C-terminal part of ghrelin. It is assumed that the number of positively charged residues in peptides correlates with their affinity to the negatively charged proteoglycans in ECM.

Long bone growth occurs at the growth plate; however, the growth plate cartilage is avascular, and the transport rate of exogenous CNPs from the blood to chondrocytes through the ECM might be a rate-limiting factor affecting their activity to accelerate bone growth [36]. Improvement of the distribution efficiency to the growth plate with controlled-release formulations might result in improvement of the efficacy of ASB20123. It also might be possible to maximize the pharmacological activity by prolonging the dosing period.

We concluded that our novel CNP analog ASB20123 might have the potential to improve growth in patients with severely short stature, such as those with achondroplasia. Controlled infusion of our novel CNP analog could not only minimize the chance of significant hypotension but also maximize its pharmacological effect on bone growth. This wide safety margin makes CNP analog suitable for treatment of growth failure and short stature.

## Supporting information

S1 Fig Amino acids sequences of CNP(1–22), human CNP-53 and ASB20123. (TIF)

S2 Fig CNP immunoreactivity in rat plasma after a single sc administration of CNP(1–22) or ASB20123 dissolved in various liquid mixtures. (TIF)

S3 Fig Body weight and body length of female ICR mice subcutaneously administered CNP(1–22) and human CNP(1–53) for 3 weeks. (TIF)

S1 Table The specific antibody titer against ASB20123 in serum of juvenile female ICR mice received ASB20123 for 8 weeks, and after 4 weeks washout period. (TIF)
