# Supplementary figures and images for: ASB20123: A novel C-type natriuretic peptide derivative for treatment of growth failure and dwarfism
Source: PLoS One. 2019 Feb 22;14(2):e0212680. doi: 10.1371/journal.pone.0212680 (PMC6386482; doi:10.1371/journal.pone.0212680)

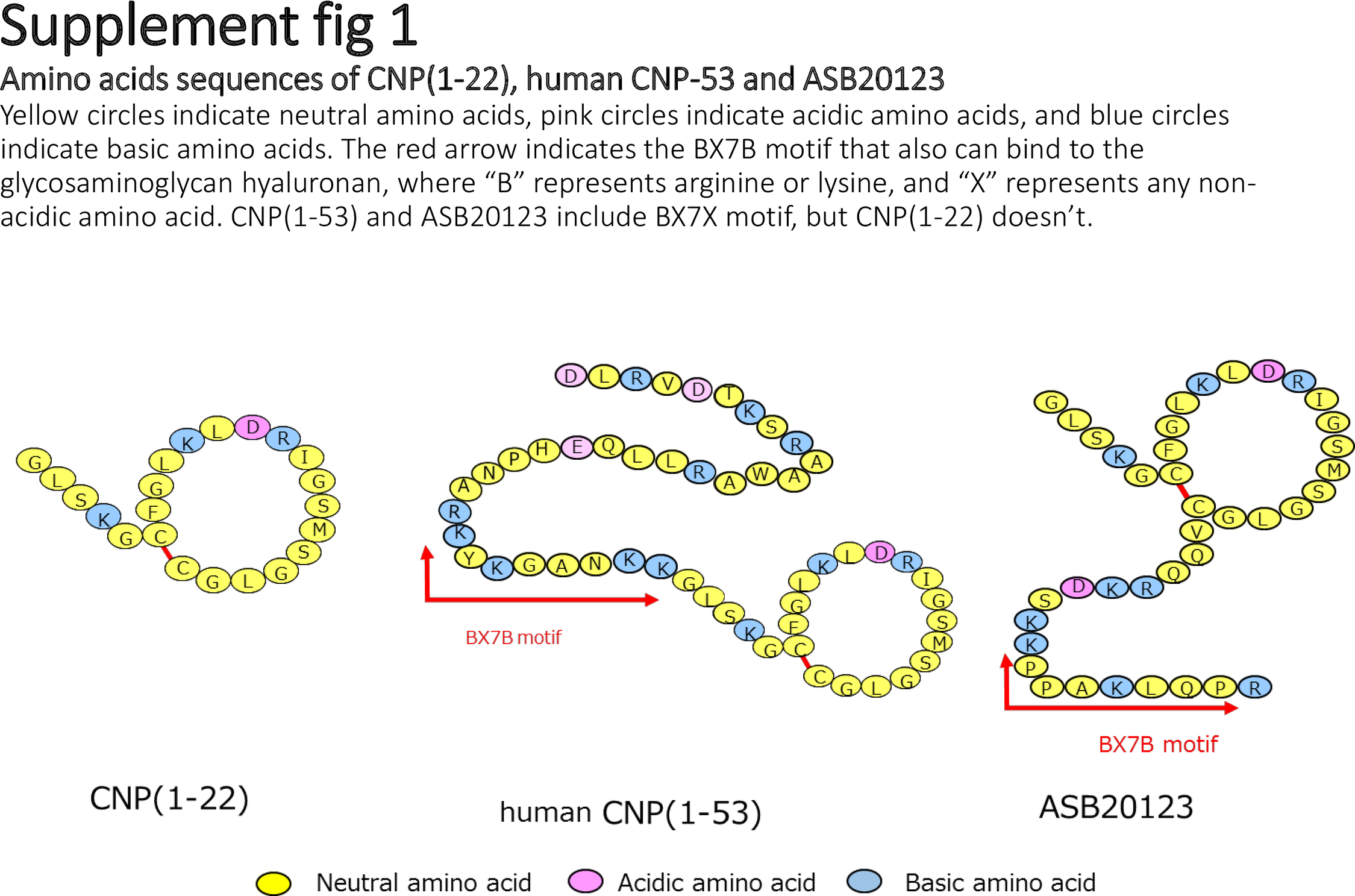

Supplement: S1 Fig — (TIF) [file pone.0212680.s001.tif]

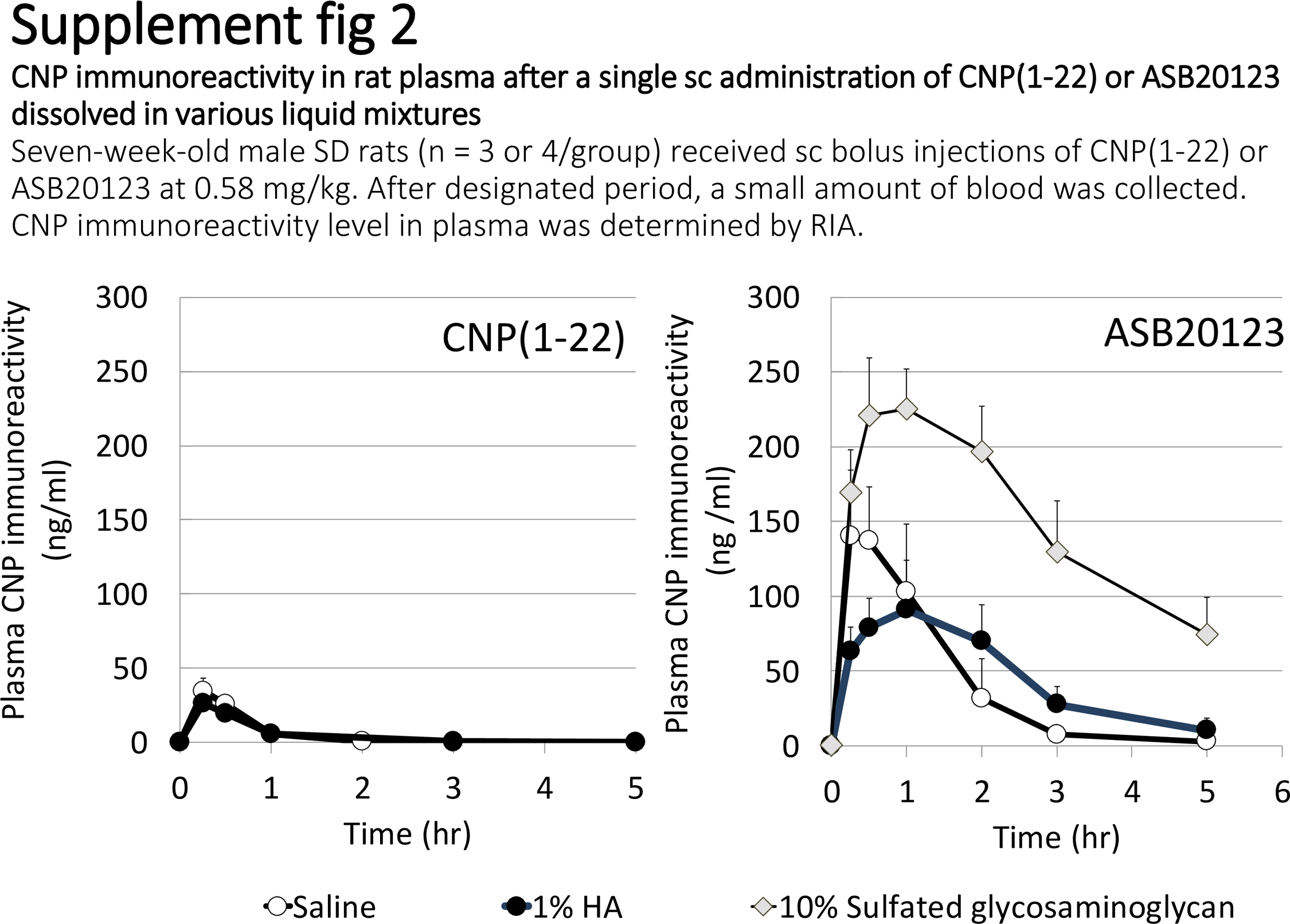

Supplement: S2 Fig — (TIF) [file pone.0212680.s002.tif]

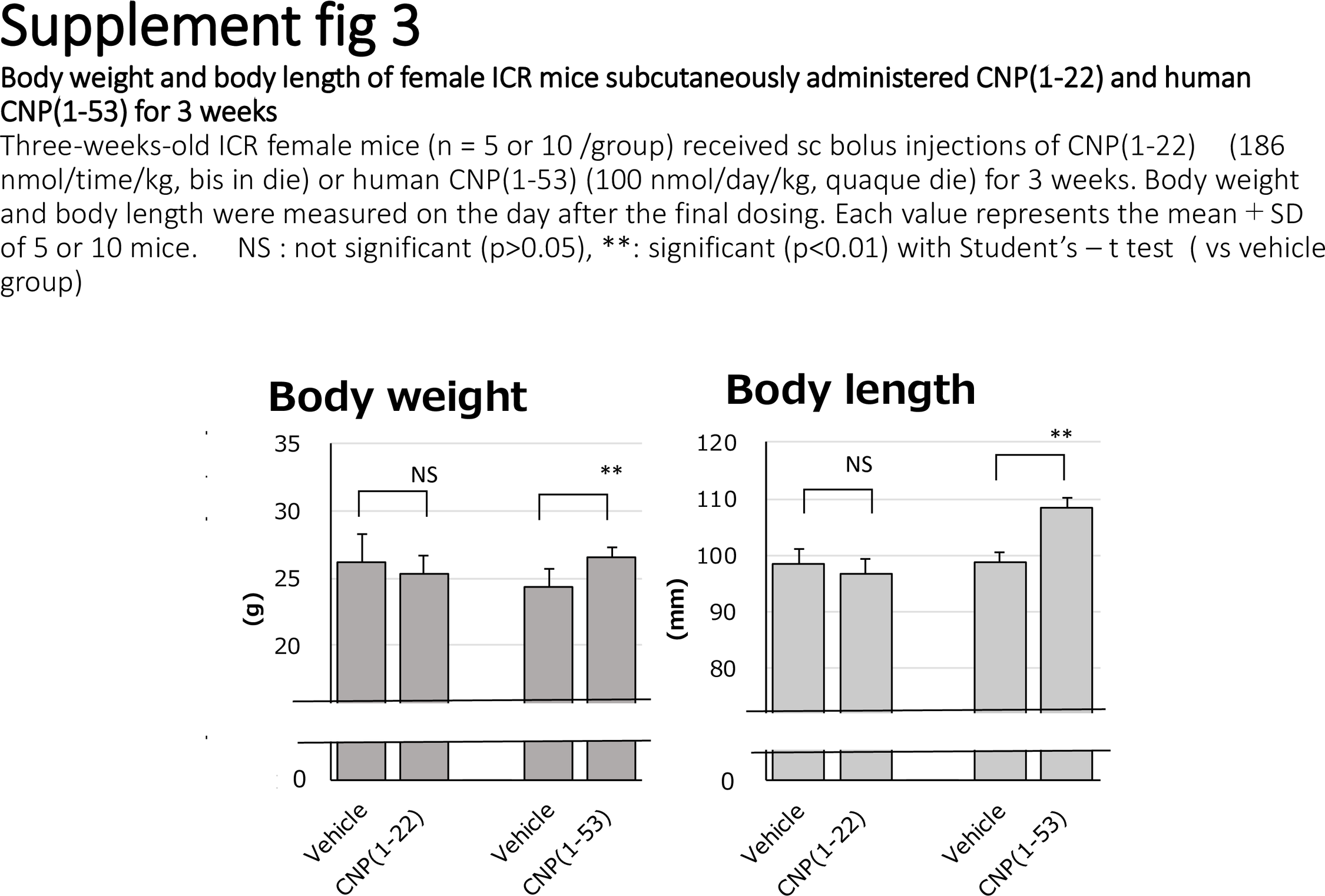

Supplement: S3 Fig — (TIF) [file pone.0212680.s003.tif]

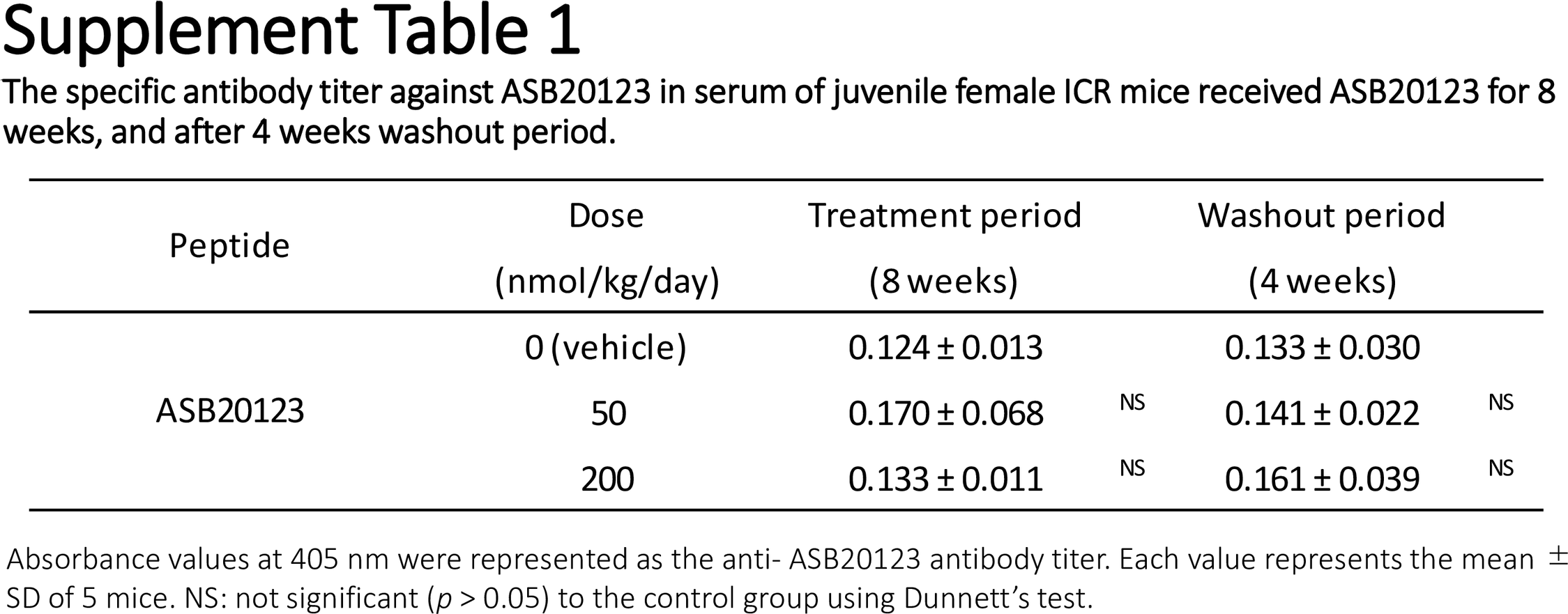

Supplement: S1 Table — (TIF) [file pone.0212680.s004.tif]
